# Transmission of *Plasmodium vivax* in South-Western Uganda: Report of Three Cases in Pregnant Women

**DOI:** 10.1371/journal.pone.0019801

**Published:** 2011-05-13

**Authors:** Mehul Dhorda, Dan Nyehangane, Laurent Rénia, Patrice Piola, Philippe J. Guerin, Georges Snounou

**Affiliations:** 1 Epicentre, Mbarara, Uganda; 2 Institut National de la Santé et de la Recherche Médicale, Unité Mixte de Recherche S 945, Paris, France; 3 Université Pierre et Marie Curie, Faculté de Médecine Pitié-Salpêtrière, Paris, France; 4 Singapore Immunology Network, Agency for Science, Technology and Research (A*STAR), Biopolis, Singapore; 5 Epicentre, Paris, France; 6 Centre for Tropical Medicine, Nuffield Department of Clinical Medicine, University of Oxford, United Kingdom; Kenya Medical Research Institute - Wellcome Trust Research Programme, Kenya

## Abstract

*Plasmodium vivax* is considered to be rare in the predominantly Duffy negative populations of Sub-Saharan Africa, as this red blood cell surface antigen is essential for invasion by the parasite. However, despite only very few reports of molecularly confirmed *P. vivax* from tropical Africa, serological evidence indicated that 13% of the persons sampled in Congo had been exposed to *P. vivax*. We identified *P. vivax* by microscopy in 8 smears from Ugandan pregnant women who had been enrolled in a longitudinal study of malaria in pregnancy. A nested polymerase chain reaction (PCR) protocol was used to detect and identify the *Plasmodium* parasites present. PCR analysis confirmed the presence of *P. vivax* for three of the women and analysis of all available samples from these women revealed clinically silent chronic low-grade vivax infections for two of them. The parasites in one woman carried pyrimethamine resistance-associated double non-synonymous mutations in the *P. vivax* dihydrofolate reductase gene. The three women found infected with *P. vivax* were Duffy positive as were nine of 68 women randomly selected from the cohort. The data presented from these three case reports is consistent with stable transmission of malaria in a predominantly Duffy negative African population. Given the substantial morbidity associated with vivax infection in non-African endemic areas, it will be important to investigate whether the distribution and prevalence of *P. vivax* have been underestimated in Sub-Saharan Africa. This is particularly important in the context of the drive to eliminate malaria and its morbidity.

## Introduction

The prevalence of *Plasmodium vivax* in tropical African became a matter for debate subsequent to the description of *P. ovale* in 1922 [1]. Indeed *P. ovale* that occurs predominantly in western and central Sub-Saharan Africa, has morphology in the blood that is difficult to distinguish from that of *P. vivax*, particularly in thick blood smears [2]. The accuracy of a *P. vivax* diagnosis in these regions was further questioned after observations in the 1930s revealed that Americans of African origin were generally innately resistant to blood infections by *P. vivax* [3], an observation that was later extended to native West Africa residents [4]. The factor responsible for this resistance was uncovered in 1976: *P. vivax* can only invade red blood cells that express the Duffy blood group antigen on their surface [5]. Thus, the perception took hold that little if any *P. vivax* transmission occurred in the predominantly Duffy negative populations, with close to 100% for West Africa, of Sub-Saharan countries (Eritrea, Djibouti, Ethiopia and Somalia excepted). It has since become customary to record any benign tertian malaria parasites originating from these regions as *P. ovale*. Nonetheless, *P. vivax* cases determined by microscopic examinations are reported from these predominantly Duffy negative populations [6].

The advent of sensitive and specific molecular techniques to detect malaria parasites opened the way to distinguish *P. ovale* from *P. vivax* confidently. The first molecularly confirmed case of *P. vivax* originating from Sub-Saharan Africa was from patients returning from the Democratic Republic of São Tomé e Príncipe, an island in the Gulf of Guinea [7], but their Duffy status could not be ascertained. This was followed by other reports of *P. vivax* infections imported from Central or Western Africa [8], [9] and Zambia [10], or acquired locally in Guinea Equatorial [11]. Recently *P. vivax* parasites were detected molecularly in mosquitoes collected in Kenya [12]. Moreover, the paradigm of the Duffy antigen dependence for *P. vivax*, was challenged by confirmed findings of *P. vivax* in the blood of Duffy negative individuals in Kenya and Brazil [12], [13], [14], and by the discovery of genetically diverse population of *P. vivax* lines capable of infecting Duffy negative populations in Madagascar [15].

Despite a relatively high prevalence of *P. vivax* in travellers returning from Sub-Saharan Africa [7], [8], the prevalence of *P. vivax* was inferred to be very low in endemic residents from these regions. Only a single case of *P. vivax* (from São Tomé and Príncipe) was detected by PCR screening in 2588 blood samples (including 851 from the Republic of Congo) from nine African malaria endemic countries [16]. However, elegant serological evidence from the Republic of Congo indicated that ca. 13% of the predominantly Duffy negative population (>95%–99%) had been exposed to *P. vivax*-infected mosquitoes [17], suggesting that significant transmission of *P. vivax* was sustainable in population predominantly insusceptible to this parasite. If this were the case, then estimates of the human population at risk from vivax malaria [6] would need to be revised upwards.

## Results

**Figure 1:**
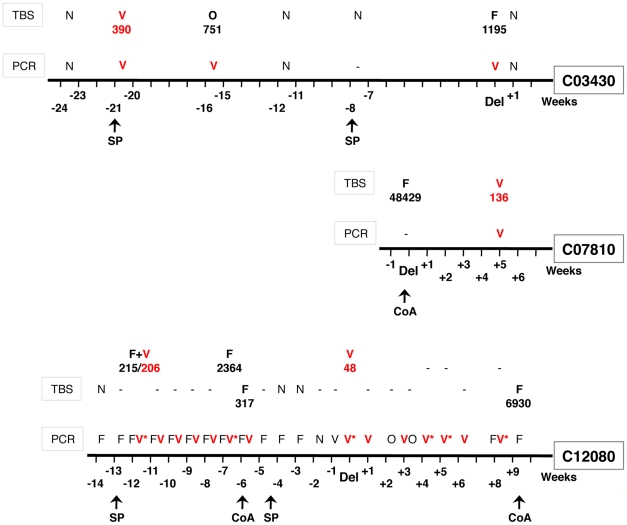
Data for the samples collected from the three women found infected with *P. vivax*. Days when treatment with artemether-lumefantrine (CoA) or IPTp using sulfadoxine-pyrimethamine (SP) was administered are indicated. Del denoted the day of delivery. Samples for PCR or thick blood smears (TBS) were only available for the weeks indicated on the time axis, and the results are presented above this axis. The parasite species identified by PCR or microscopy are provided, as is the average parasitaemia (parasites per µl of blood) recorded for each species: F = *P. falciparum*; V = *P. vivax* (shown in red); O = *P. ovale*; N = negative sample, and “-“ denotes a missing sample. The patient codes provided are those internally and randomly assigned upon recruitment. Samples from patient C012080 from which the *Pvdhfr* fragment could be amplified and sequenced are indicated by an asterisk (*).

*P. vivax* was identified in thick blood smears (TBS) from eight of the women in a cohort of pregnant women that were being followed-up as part of a study of malaria in pregnancy. For all the smears, parasitaemias were very low (<800 parasite per µl of blood, i.e. with a maximum of 50 parasite forms observed per 500 white blood cells). DNA was extracted from the blood samples corresponding to these slides and analyzed by PCR. Three samples were negative by amplification. This discrepancy, which was unlikely to be due to low PCR assay sensitivity as this was monitored throughout the study, was most probably due to the combined facts that the blood samples were collected on filter paper two or more years prior to template preparation (long storage is associated with DNA degradation) and that the parasitaemias were close to the limits of microscopic detection. Moreover, the template added to each PCR reaction corresponded to a small volume of blood, close to that scanned for thick smear examination, which makes it likely for the PCR analysis to fail to detect the parasite from such samples. In two other samples, *P. falciparum*, alone or as a mixed infection with *P. ovale*, was detected. For the remaining three samples the presence of *P. vivax* was confirmed molecularly. DNA was then purified from all the blood samples collected from these three patients during their follow-up, and subjected to PCR analysis. The PCR results and the TBS readings for these patients are presented schematically (Fig. 1). The sensitivity of parasite detection by PCR was expected to be higher for samples from C12080 (n = 23) as compared that that from samples from C03430 (n = 6) and C07810 (n = 6), because only dried blood spots were available from the last two, whereas frozen RBC pellets were available for the first. Indeed, the DNA template screened from dried blood spots corresponded to ca. 1 µl of blood while that from frozen blood pellets corresponded to 10 µl, probably the reason for the large number of *Plasmodium*-positive samples from C12080 (Fig. 1). For the whole set, only 15 samples were analyzed both by TBS and PCR, and a single case of microscopic misdiagnosis was identified, namely on the day of delivery for C03430 (Fig. 1). This could be due to the presence of very early *P. vivax* ring stages, where the characteristic RBC enlargement and Schüffner dots are yet to develop, making these forms particularly easily confused with *P. falciparum* early rings in thick blood smears.

The paucity of parasites in our samples made it difficult to genotype satisfactorily the *P. vivax* parasites in the various samples, thus it was not possible to establish whether the *P. vivax* populations observed at different times in the same woman or in different women were genetically related. Nonetheless, amplification of the fragment spanning a large portion of *Pvdhfr* was successful for some of the samples collected from patient C12080 (Fig. 1), as this was considered important given the observation that *P. vivax* was detected in the blood samples collected over the weeks following administration of sulfadoxine-pyrimethamine (SP) as apart of intermittent preventive therapy in pregnancy (IPTp). As compared to the wild-type gene (from the Sal I line), the fragments from all the samples shared the two non-synonymous mutations at residues 58 (S to R) and 117 (S to N). These mutations have been associated with resistance to pyrimethamine [18]. Non-synonymous mutations at other residues, and in particular 57 and 61, were not observed.

The Duffy status of the three women with *P. vivax* infections was determined by RFLP-PCR to be heterozygous positive Fy(b+,b−). In order to estimate the prevalence of Duffy positivity in the cohort, 68 randomly selected women were also genotyped. Eight were Duffy positive heterozygous Fy(b+,b−), one was Duffy positive homozygous Fy(a+,b+), and the remainder were Duffy negative Fy(b−,b−). Thus, a prevalence of 13.2% (95% CI 6.6–24.1) was estimated for Duffy positivity among the pregnant women.

## Discussion

The data we present constitutes the first set of longitudinal observations of molecularly confirmed *P. vivax* infections in pregnant native Ugandan residents. Despite being confined to three persons, the observations allow formulating some conclusions and speculations. It is likely that *P. vivax* in Mbarara District is transmitted in a stable manner because the three women harbouring this parasite in their blood were recruited many months apart (May 2007 for C03430, November 2007 for C07810, and December 2008 for C12080). This is consistent with the conclusion derived from the studies conducted in Pointe-Noire, Republic of Congo, namely that *P. vivax* transmission could be maintained by a minority of Duffy positive (hetero- or homozygous) individuals [17]. In a previous study, PCR analysis failed to reveal the presence of *P. vivax* in 2588 blood samples collected from 9 Sub-Saharan African countries, including 851 from the Republic of Congo [16]. This might simply reflect the fact that most of the DNA templates were extracted from archival blood dried on filter paper, and might have corresponded to a small volume of blood (<1 µl), which would reduce the sensitivity for the detection of sub-microscopic infections. Alternatively, it might be due to the lower frequencies of Duffy positive individuals in these countries (<5%), though this would seem to contradict the high proportion of persons (13%) who have been exposed to *P. vivax*-infected mosquito bites [17], which implies a relatively high proportion of *P. vivax* carriers in the community.

It should be noted that the three pregnant women had not reported any clinical symptoms on the days when the blood samples analysed were found to be positive for *P. vivax* by PCR analysis. This is consistent with the fact that the *P. vivax* parasitaemias were in most cases below the threshold of microscopic detection. Such low-grade chronic infections might indicate a certain level of immunity to *P. vivax*, and/or might be due to a reduction of erythrocyte susceptibility to *P. vivax* in Duffy negative heterozygotes [19]. Nonetheless, the fact that the infections were detected in pregnant women is a cause for concern, because *P. vivax* during pregnancy has been shown to be associated with maternal anaemia and low birth-weight [20]. In this context, the finding of *Pvdhfr* mutations associated with resistance to pyrimethamine is noteworthy, as it might indicate that the selection of pyrimethamine-resistant *P. vivax* has been underway in Uganda for some time. Whether the failure of SP treatment to clear *P. vivax* (Fig. 1) was due to these mutations or the shorter half-life of both drugs in pregnant women [21] remains to be determined.

As it stands, the prevalence of malaria in the residents of Mbarara District cannot be estimated from samples collected only from a cohort of pregnant women. These samples are further biased by the fact that a subset of these women was specifically recruited for a study of drug efficacy against *P. falciparum* infection [22]. *P. falciparum* remains by far the dominant species observed by microscopy in Uganda. This is also the case for the samples collected from the pregnant women in our cohort. For example of the 3810 blood smears examined in 2008, *P. falciparum* was detected alone in 196 smears, whereas *P. ovale* and *P. vivax* were observed alone in only 4 and 2 smears, respectively. Mixed species infections, in all cases with *P. falciparum*, were found in 7 of these smears (3 with *P. malariae*, and 4 with *P. vivax*). Given that several thousand samples were collected from the women of this cohort, a large-scale PCR analysis of all the samples available was precluded on grounds of cost. Nonetheless, in a concurrent longitudinal study evaluating rapid diagnostic tools for malaria in a subset of 96 pregnant women from the same cohort, 484 samples were collected and PCR analysis revealed 79 samples with pure *P. falciparum* infections, and only 2 with *P. malariae* and 1 with *P. ovale*, but none with *P. vivax*. Nonetheless, it is unlikely that the *P. vivax* prevalence in the community would be negligible. First, the percentage of Duffy positive individuals (13%) is relatively high. Second, in two of the women *P. vivax* was repeatedly detected by PCR in blood samples collected during follow-up (either due to recrudescences/relapses or to reinfections). Such a chronic infection constitutes a sustained reservoir of infection that would increase the potential for transmission. At present, it is not known whether pregnant women in Africa are more susceptible to *P. vivax* than others in the community, as is the case for those in India [23] or in Thailand [24]. This will have to await PCR analysis of samples collected from the community at large.

In conclusion, we present evidence consistent with stable transmission of *P. vivax* infections sustained in a predominantly Duffy negative population in south-western Uganda. These observations lend credence to previous reports of *P. vivax* infections in residents of western and central Africa, and explain the relatively high frequencies of *P. vivax* imported with travellers returning from these regions. If the *P. vivax* infections in sub-Saharan Africa are generally low grade and asymptomatic, then it is likely that the prevalence of *P. vivax* will be inaccurate when based only on results from microscopic examination of smears collected for passive case detection. Clearly further detailed investigations of the epidemiology of *P. vivax* in the largely Duffy negative Sub-Saharan African populations are warranted. It might lead this species to be taken into consideration when planning for malaria elimination in Sub-Saharan Africa. In this context, the use of primaquine, the only drug available to eliminate *P. vivax* hypnozoites, poses a particular challenge in an African setting where the prevalence of glucose-6-phosphate deficient inhabitants severely limits its deployment.

## Materials and Methods

### Cohort, sample collection

A cohort of African Ugandan pregnant women (n = 1229) within a radius of 15 km from Mbarara town in south-western Uganda was recruited between October 2006 and January 2009 for investigations of malaria in pregnancy and its treatment [22], with blood samples collected periodically for blood smears and dried blood spots on filter paper (Whatman 3 MM). A sub-cohort was also followed with weekly blood samples collected by fingerprick, or by venepuncture if other tests were being performed [22], with EDTA as anticoagulant.

Blood samples were used to prepare blood smears, filter paper spots or to separate red blood cells (RBCs). An aliquot of the blood collected on EDTA was centrifuged to obtain a RBC pellet that was stored at −80°C until further analysis.

### Microscopy

Two hundred high power fields (1000×) of each Giemsa-stained thick blood smear (TBS) were read before declaring the slide negative. Positive slides and 10% of all negative slides were examined by two independent microscopists for the purposes of internal quality control, and a third read was performed in cases of discordance (positive/negative, parasitaemia variation >50%, and species). Average parasitaemia from the two closest results was estimated by counting parasites against 200 white blood cells (a figure increased to 500 if ≤9 parasites were observed initially) and by assuming an average WBC count of 8000 per µL.

### Molecular analysis

The presence and identity of the four *Plasmodium* species that infect humans in Africa was established using a nested PCR protocol [25], with minor modification to allow detection of the two *P. ovale* types [26]. The primary PCR reaction was initiated either: 1) with DNA extracted from the filter spots on which the finger prick blood was dried using commercial kits (Qiagen DNAMicro, Qiagen, Germany); or 2) directly on a pellet derived from a 5 µl aliquot of the frozen RBCs (equivalent to 10 µl of whole blood), obtained by centrifugation of a lysate of these cells after suspension in phosphate buffered saline (PBS) supplemented with 0.05% w/v saponin and a further centrifugation step to recover the pellet after washing in PBS [27].

A fragment of the *Pvdhfr-ts* gene was amplified as by nested PCR as previously described [18], using the primers VDT-OF (5′-ATGGAGGACCTTTCAGATGTATTTGACATT) + VDT-OR (5′-GGCGGCCATCTCCATGGTTATTTTATCGTG) for the primary reactions, and VDT-OF + VDT-LiF (5′-CTTCCCCGAGTTTGACGAAAGCCAGTTTCG) for the secondary reaction. The amplified fragment was cloned into a pCR4-Topo vector (Invitrogen, The Netherland), sequenced commercially, and the sequences assembled and analyzed using the Lasergene suite of programs (DNASTAR Inc, USA).

The Duffy status was determined based on restriction fragment length polymorphism (RFLP) of two PCR fragments as described previously [19], [28] obtained by nested PCR using FY277 (5′-CAGGAAGACCCAAGGCCAG) + *FY*851 (5′-GGCCAAGACGGGCACCACAATG) for the primary reaction and FY277 + *FY*Pdn (5′-CCATGGCACCGTTTGGTTCAGG) or *FY*851 + *FY*up (5′-GACTCTTCCGGTGTAACTCTGATG) for the secondary reaction. The amplified fragments were then digested with the appropriate restriction enzymes and product sizes were determined after agarose gel electrophoresis.

### Ethical approval

The clinical samples examined for this report were collected in a study conducted according to the Declaration of Helsinki. The study protocol for the sub-cohort and the forms used to document informed written consent were submitted to and approved by three ethics committees – two from the Mbarara University of Science and Technology and one from the Ugandan National Council for Science and Technology. The research protocol for the main cohort was also approved by these ethics review committees as well as by the “Comité de Protection des Personnes” (Ile-de-France XI, France).
